# A preliminary study of bullying on University of Nursing in Taiwan

**DOI:** 10.1093/eurpub/ckac131.428

**Published:** 2022-10-25

**Authors:** M Hsieh

**Affiliations:** Nursing Institute, Hung Kuang University, TaiChung, Taiwan

## Abstract

**Background:**

According to the COVID-19 pandemic progress, the increase in online learning and communication, the survey shows that 36.3% of children have been bullied online (Children’s Welfare Alliance, 2021). However, campus bullying has long-term effects on students’ physiology and psychology, and will also extend to society in the future to form a social problem.

**Aims:**

It is generally believed that university students are relatively mature and have more freedom in taking courses. They believe that there are few bullying incidents that need attention on university campuses. Therefore, to understand the patterns of bullying on university campuses of nursing in Taiwan through this study.

**Methods:**

Referring to the definition and selecting the published within good reliability and validity of ‘University Students’ Campus Life Experience and Interpersonal Interaction Experience Survey', the subjects in domestic university campuses choose the closest answer according to their own situation.

**Results:**

In a total of 2570 valid questionnaires, the highest incidence of verbal bullying (38%), and those who don't want to answer gender “experienced bullying in person” (42.9%). Gender was shown to be statistically significant in bullying experience (p=.017). It shows that university students of nursing in Taiwan have obvious room for improvement in gender issues.

**Conclusions:**

Through this study, it is known that campus bullying exists in different types of experiences on university campuses of nursing in Taiwan. The bullying impact is often not only in school, and the impact will even extend to enter social work. Faculties must also be the gatekeepers of bullying prevention, but in fact, even senior faculties may not be very clear about what bullying is, how to properly handle bullying incidents, and how to prevent bullying, so every faculty should have the ability to take appropriate, fast and effective treatment when bullying occurs.

**Key messages:**

• Campus bullying has long-term effects on students’ physiology and psychology, and will also extend to society in the future to form a social problem.

• Verbal bullying (38%) had the highest incidence, those who don’t want to answer gender “experienced bullying in person” (42.9%).

